# Twelve lateral flow immunoassays (LFAs) to detect SARS-CoV-2 antibodies

**DOI:** 10.1016/j.jinf.2021.12.007

**Published:** 2022-03

**Authors:** Sophie I. Owen, Christopher T. Williams, Gala Garrod, Alice J. Fraser, Stefanie Menzies, Lisa Baldwin, Lottie Brown, Rachel L. Byrne, Andrea M. Collins, Ana I. Cubas-Atienzar, Margaretha de Vos, Thomas Edwards, Camille Escadafal, Daniela M. Ferreira, Tom Fletcher, Angela Hyder-Wright, Grant A. Kay, Konstantina Kontogianni, Jenifer Mason, Elena Mitsi, Tim Planche, Jilian A. Sacks, Joseph Taylor, Stacy Todd, Caroline Tully, Luis E. Cuevas, Emily R. Adams

**Affiliations:** a Centre for Drugs and Diagnostics Research, Liverpool School of Tropical Medicine (LSTM), Liverpool, United Kingdom; b Liverpool University Hospitals NHS Foundation Trust, Liverpool, United Kingdom; c FIND, Geneva, Switzerland; d Department of Clinical Sciences, Liverpool School of Tropical Medicine (LSTM), Liverpool, United Kingdom; e Liverpool Clinical Laboratories, Liverpool University Hospitals NHS Foundation Trust, Liverpool, United Kingdom; f Institute for Infection and Immunity, St George's University of London, London, United Kingdom; g St George's University Hospitals NHS Foundation Trust, London, United Kingdom

**Keywords:** SARS-CoV-2, Lateral flow immunoassays, COVID-19, IgM, IgG

## Abstract

**Background:**

There are an abundance of commercially available lateral flow assays (LFAs) that detect antibodies to SARS-CoV-2. Whilst these are usually evaluated by the manufacturer, externally performed diagnostic accuracy studies to assess performance are essential. Herein we present an evaluation of 12 LFAs.

**Methods:**

Sera from 100 SARS-CoV-2 reverse-transcriptase polymerase chain reaction (RT-PCR) positive participants were recruited through the FASTER study. A total of 105 pre-pandemic sera from participants with other infections were included as negative samples.

**Results:**

At presentation sensitivity against RT-PCR ranged from 37.4 to 79% for IgM/IgG, 30.3–74% for IgG, and 21.2–67% for IgM. Sensitivity for IgM/IgG improved ≥ 21 days post symptom onset for 10/12 tests. Specificity ranged from 74.3 to 99.1% for IgM/IgG, 82.9–100% for IgG, and 75.2–98% for IgM. Compared to the EuroImmun IgG enzyme-linked immunosorbent assay (ELISA), sensitivity and specificity ranged from 44.6 to 95.4% and 85.4–100%, respectively.

**Conclusion:**

There are many LFAs available with varied sensitivity and specificity. Understanding the diagnostic accuracy of these tests will be vital as we come to rely more on the antibody status of a person moving forward, and as such manufacturer-independent evaluations are crucial.

## Introduction

As of May 2021, there have been over 100,000,000 confirmed cases of COVID-19 worldwide, however the total number of cases is much higher.^1^ This is due to, amongst other reasons, the lack of diagnostic testing worldwide in the first wave of the pandemic, the continued difficulties in testing in some lower-middle income countries and the number of asymptomatic infections that continue to go undetected.^2^^,^^3^ Detecting antibody responses to SARS-CoV-2 therefore could prove vital, both for understanding previous exposure on an individual level, but also at community and regional levels.

During SARS-CoV-2 infection, IgM and IgG titres begin to increase from around 10 days post-symptom onset with IgM titres generally declining earlier than IgG.^4^^,^^5^ The reference standard for detecting an antibody response, either IgM, IgG, or both, to SARS-CoV-2 is the immunoassay, usually either an enzyme-linked immunosorbent assay (ELISA) or chemiluminescence assay. Many immunoassays have been developed and commercialised for SARS-CoV-2 which are highly sensitive and specific.^6^ The process however is time-consuming, expensive, requires specialist laboratory equipment and trained professionals to perform.

To this end, large numbers of lateral-flow immunoassays (LFAs) have been developed that detect IgM and IgG responses. These are simple tests which require only a drop of blood and the addition of buffer to give a result in 10–15 min. Results are easy to interpret; if a test line appears the participant is considered positive, if no test line appears the participant is considered negative. LFAs are easy to mass-produce and are affordable making them ideal for mass-testing of populations, rapid identification of antibody response in travel situations, or for home-testing. Those that detect both IgM and IgG are also able to give an indication of when that person was likely infected, due to the dynamics of the antibody response.

Many LFAs are commercially available, and externally performed diagnostic accuracy studies, independent of the manufacturer, are required to generate robust performance data. Here, we present the evaluation of twelve LFAs and describe their diagnostic accuracy in a cohort of 100 confirmed SARS-CoV-2 positive participants with varying disease severity and 105 samples from participants confirmed as negative or collected pre-pandemic. This study forms part of a larger initiative to generate and share independent performance data on COVID-19 tests coordinated by FIND, the global diagnostics alliance.

## Methods

### Ethics statement

Research samples were provided with informed written consent. Participants were recruited through the Facilitating. A SARS CoV-2 TEst for Rapid triage (FASTER) study, approved by the National Health Service Research Ethics Committee (20/SC/0169) under the Integrated Research Application System no. 282,147.

### Participants

A total of 142 serum/plasma samples from 125 SARS-CoV-2 RT-qPCR -positive participants were used in this study to assess the sensitivity of the LFAs. One hundred serum/plasma samples from RT-qPCR -positive participants were used for each LFA due to the limited quantity of some serum samples. Full details are given in Table S1. Briefly, 24 patients presenting at the Liverpool University Hospitals NHS Foundation Trust (Liverpool, UK) were recruited as part of the FASTER study and provided 41 serum samples collectively at different timepoints (D0, D2, D7, D28 post-admission). Sera from patients with RT-qPCR confirmed SARS-CoV-2 infection (*n* = 84) were provided by Liverpool Clinical Laboratories (LCL) as leftover diagnostic samples. Participants with RT-qPCR confirmed SARS-CoV-2 infection who did not result in hospital attendance (*n* = 12) were also recruited^7^. In addition, the NIBSC COVID-19 convalescent plasma panel, human (20/118), as well as NIBSC 20/130 plasma positive control were used for the evaluation. The COVID-19 convalescent plasma panel (NIBSC 20/118) and NIBSC 20/130 were obtained from the National Institute for Biological Standards and Control, UK.

A total of 105 SARS-CoV-2 negative serum samples were used to assess specificity of the LFAs. See Table S2 for full details. These consisted of 84 pre-pandemic sera collected from individuals diagnosed with influenza A (*n* = 20), tuberculosis (TB) (*n* = 10), human immunodeficiency virus (HIV) (*n* = 10), TB/HIV (*n* = 10), dengue virus (*n* = 10), parasitic diseases (*n* = 12), human coronavirus 229E (*n* = 10) and human coronavirus OC43 (*n* = 2). A panel of pre-pandemic plasma from participants with non-COVID-19-related fever (*n* = 20) were provided by FIND and an additional quality assurance sample (*n* = 1).

### Enzyme-linked immunosorbent assay (ELISA) to detect IgG

The Anti-SARS-CoV-2 ELISA (IgG) kit (EI 2606–9601 G) (EuroImmun, Germany) was used to screen all serum samples for the presence of anti-SARS-CoV-2 IgG, as per the manufacturer's instructions. Samples with an OD value greater than the calibrator were considered positive, samples with OD value lower than the calibrator were considered negative.

### Lateral flow immunoassays

Twelve LFAs (Table 1) were evaluated according to manufacturer's instructions. Briefly, 10–20 µl serum was required depending on the test, followed by 2–3 drops of buffer. Results were read independently by two people; if there was any disagreement a third person acted as a tiebreaker. Full details are given in Table 1. Of the 12 tests, 11 detected IgM and IgG separately, with only Beijing Wantai giving a ‘total antibody’ result. All tests were CE-IVD marked.

**Table 1:** Details of LFAs evaluated.

Manufacturer	Test name (manufacturer)	Referred to herein as	Product Code	Lot Numbers	Volume of sera (µl)	Drops of buffer	Time to result (minutes)
Beijing Wantai Biological Pharmacy Enterprise Co., Ltd	WANTAI SARS-CoV-2 Ab Rapid Test	Beijing Wantai	WJ-2750	JNB20200408	10	2	15
Bionote Co., LTD.	NowCheck COVID-19 IgM/IgG Test	Bionote	RB2901DG	2901D002	10	3	10
Core Technology Co., Ltd	COVID-19 IgM/IgG Ab Test	Core Technology	B290–21	20,200,406	10	2	10
CTK Biotech	Onsite COVID-19 IgM/IgG Rapid Test	CTK Biotech	R0180C	F0507R1C00	10	2	10
Edinburgh Genetics Limited	Edinburgh Genetics COVID-19 Colloidal Gold Immunoassay Testing Kit, IgM/IgG Combined	Edinburgh Genetics	TIL225AEGCV0055	2000555A	20 into 2 ml buffer	2–3	10
GenBody Inc.	COVID-19 IgM/IgG	GenBody	COVI040, PQGB021 (reader)	FJF029201	10	3	10
Jiangsu Bioperfectus Technologies Co., Ltd	Novel Corona Virus(SARS-CoV-2)IgM/IgG Rapid Test Kit	Jiangsu Bioperfectus	SC30201W	20,200,401	10	3	10
PRIME4DIA Co., Ltd	P4DETECT COVID-19 IgM/IgG	PRIME4DIA		CMG200701	10	3	10
Qingdao HIGHTOP Biotech Co., Ltd.	SARS-CoV-2 IgM/IgG Ab Rapid Test	Qingdao HIGHTOP	H100	COV1252004C	10	2	15
Shanghai Kehua Bio-Engineering Co., Ltd	Diagnostic Kit for SARS-CoV-2 IgM/IgG Antibody (Colloidal Gold)	Shanghai Kehua	R-423–20-C-CE	423,200,334	10	3	15
Shenzhen Bioeasy Biotechnology Co., Ltd	2019-Novel Coronavirus (2019-nCoV) IgM/IgG GICA Rapid Test Kit	Shenzhen Bioeasy	YRLG22301025	2003N104	10	2	10
Zhuhai Livzon Diagnostics Inc.	Diagnostic Kit for IgM/IgG Antibody to Coronavirus (SARS-CoV-2) (Lateral Flow)	Zhuhai Livzon		CK2004240410	10	2	10

### Data analysis

Sensitivity was calculated against RT-qPCR confirmed SARS-CoV-2 infections including sensitivity when stratified by days post-symptom onset. Specificity was calculated against RT-qPCR confirmed SARS-CoV-2 negative samples or samples collected pre-pandemic. Sensitivity was then calculated against RT-qPCR confirmed SARS-CoV-2 infections also positive by IgG ELISA. Specificity was calculated against IgG ELISA negative samples. Percentage agreement and Cohen's Kappa statistic against IgG ELISA were calculated.^8^ Data analyses were carried out in MedCalc for Windows, version 19.8 (MedCalc Software, Ostend, Belgium).

## Results

### Sensitivity and specificity against RT-qPCR

**Fig. 1:**
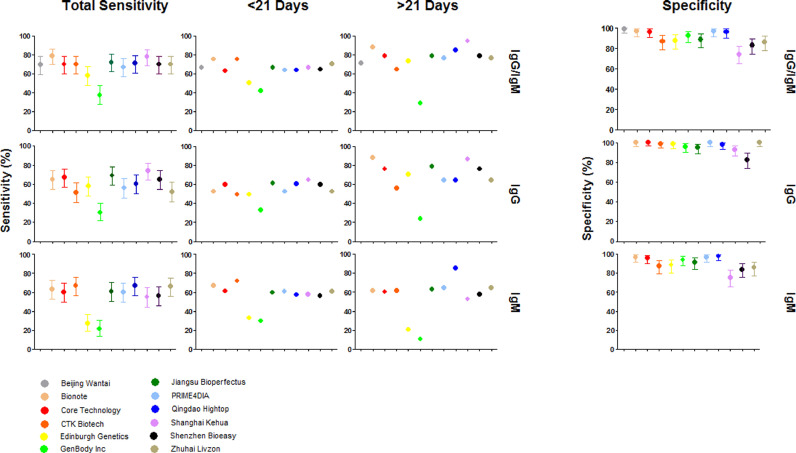
Sensitivity (total, ≤ 21 and > 21 days post symptom onset) and specificity of the antibody response (IgM/IgG, IgG, IgM) to each LFA. Note, sensitivity was calculated against RT-PCR results and specificity was calculated using a pre-pandemic panel.

Sensitivity of the LFAs against RT-qPCR ranged from 37.4 to 79.0% for IgM/IgG, 30.3–74.0% for IgG only, and 21.2–67.0% for IgM only (Table 2, Fig. 1). The sensitivity for an IgM/IgG response increased in 10 out of 12 tests at > 21 days post-symptom onset, with a mean increase of 15.0% (Table 2, Fig. 1). The sensitivity for IgG increased > 21 days post-symptom onset, with a mean increase of 16.5% (Table 2, Fig. 1). For IgM, sensitivity was higher ≤ 21 days post-symptom onset in six of the LFAs and higher at > 21 days post-symptom onset for five tests (Table 2, Fig. 1).

**Table 2:** Sensitivity and specificity of the 12 LFAs. Sensitivity was calculated using SARS-CoV-2 RT-qPCR positive sera/plasma and specificity determined on pre-pandemic sera/plasma.

Test	Ig	All samples		≤ 21 DAYS POST SYMPTOM ONSET Sensitivity vs. RT-qPCR (%)	> 21 DAYS POST SYMPTOM ONSET Sensitivity vs RT-qPCR (%)	More sensitive > 21 days?
		Sensitivity vs RT-qPCR (%) [95% CI]	Specificity vs pre-Pandemic panel (%) [95% CI]			
Beijing Wantai	IgG + IgM	69.7 (69/99 TP) [59.7–78.5]	99.1 (104/105 TN) [94.8–100]	66.7 (38/57)	71.1 (27/38)	Yes
Bionote	IgG + IgM	79.0 (79/100 TP) [69.7–86.5]	97.0 (97/100 TN) [91.5–99.4]	75.4 (46/61)	88.2 (30/34)	Yes
	IgG	65.0 (65/100 TP) [54.8–74.3]	100 (100/100 TN) [96.4–100]	52.5 (32/61)	88.2 (30/34)	Yes
	IgM	63.0 (63/100 TP) [52.8–72.4]	97.0 (97/100 TN) [91.5–99.4]	67.2 (41/61)	61.8 (21/34)	No
Core Technology	IgG + IgM	70.0 (70/100 TP) [60.0–78.8]	96.2 (101/105 TN) [90.5–99.0]	63.2 (36/57)	79.0 (30/38)	Yes
	IgG	67.0 (67/100 TP) [56.9–76.1]	100 (105/105 TN) [96.6–100]	59.7 (34/57)	76.3 (29/38)	Yes
	IgM	60.0 (60/100 TP) [49.7–69.7]	96.2 (101/105 TN) [90.5–99.0]	61.4 (35/57)	60.5 (23/38)	No
CTK Biotech	IgG + IgM	70.0 (70/100 TP) [60.0–78.8]	86.7 (85/98 TN) [78.4–92.7]	75.4 (46/61)	64.7 (22/34)	No
	IgG	51.0 (51/100 TP) [40.8–61.1]	99.0 (97/98 TN) [94.5–100]	49.2 (30/61)	55.9 (19/34)	Yes
	IgM	67.0 (67/100 TP) [56.9–76.1]	87.8 (86/98 TN) [79.6–93.5]	72.1 (44/61)	61.8 (21/34)	No
Edinburgh Genetics	IgG + IgM	58.0 (58/100 TP) [47.7–67.8]	87.6 (85/97 TN) [79.4–93.4]	50.8 (31/61)	73.5 (25/34)	Yes
	IgG	56.0 (56/100 TP) [45.7–65.9]	99.0 96/97 TN) [94.4–100]	49.2 (30/61)	70.6 (24/34)	Yes
	IgM	27.0 (27/100 TP) [18.6–36.8]	88.7 (86/97 TN) [80.6–94.2]	32.8 (20/61)	20.6 (7/34)	No
GenBody Inc.	IgG + IgM	37.4 (37/99 TP) [27.9–47.7]	92.4 (97/105 TN) [85.5–96.7]	42.1 (24/57)	29.0 (11/38)	No
	IgG	30.3 (30/99 TP) [21.5–40.4]	96.2 (101/105 TN) [90.5–99.0]	33.3 (19/57)	23.7 (9/38)	No
	IgM	21.2 (21/99 TP) [13.6–30.6]	94.3 (99/105 TN) [88.0–97.9]	29.8 (17/57)	10.5 (4/38)	No
Jiangsu Bioperfectus	IgG + IgM	72.0 (72/100 TP) [62.1–80.5]	88.6 (93/105 TN) [80.9–94.0]	66.7 (38/57)	79.0 (30/38)	Yes
	IgG	69.0 (69/100 TP) [59.0–77.9]	95.2 (100/105 TN) [89.2–98.4]	61.4 (35/57)	79.0 (30/38)	Yes
	IgM	61.0 (61/100 TP) [50.7–70.6]	91.4 (96/105 TN) [84.4–96.0]	59.7 (34/57)	63.2 (24/38)	Yes
PRIME4DIA	IgG + IgM	67.0 (67/100 TP) [56.9–76.1]	97.0 (98/101 TN) [91.6–99.4]	63.9 (39/61)	76.5 (26/34)	Yes
	IgG	56.0 (56/100 TP) [45.7–65.9]	100 (101/101 TN) [96.4–100]	52.5 (32/61)	64.7 (22/34)	Yes
	IgM	60.0 (60/100 TP) [49.7–69.7]	97.0 (98/101 TN) [91.6–99.4]	60.7 (37/61)	64.7 (22/34)	Yes
Qingdao HIGHTOP	IgG + IgM	71.0 (71/100 TP) [61.1–79.6]	96.0 (97/101 TN) [90.2–98.9]	63.9 (39/61)	85.3 (29/34)	Yes
	IgG	60.0 (60/100 TP) [49.7–69.7]	98.0 (99/101 TN) [93.0–99.8]	60.7 (37/61)	64.7 (22/34)	Yes
	IgM	67.0 (67/100 TP) [56.9–76.1]	98.0 (99/101 TN) [93.0–99.8]	57.4 (35/61)	85.3 (29/34)	Yes
Shanghai Kehua	IgG + IgM	78.0 (78/100 TP) [68.6–85.7]	74.3 (78/105 TN) [64.8–82.3]	66.7 (38/57)	94.7 (36/38)	Yes
	IgG	74.0 (74/100 TP) [64.3–82.3]	93.3 (98/105 TN) [86.8–97.3]	64.9 (37/57)	86.8 (33/38)	Yes
	IgM	55.0 (55/100 TP) [44.7–65.0]	75.2 (79/105 TN) [65.9–83.1]	57.9 (33/57)	52.6 (20/38)	No
Shenzhen Bioeasy	IgG + IgM	70.0 (70/100 TP) [60.0–78.8]	82.9 (87/105 TN) [74.3–89.5]	64.9 (37/57)	79.0 (30/38)	Yes
	IgG	65.0 (65/100 TP) [54.8–74.3]	82.9 (87/105 TN) [74.3–89.5]	59.7 (34/57)	76.3 (29/38)	Yes
	IgM	56.0 (56/100 TP) [45.7–65.9]	83.8 (88/105 TN) [75.4–90.3]	56.1 (32/57)	57.9 (22/38)	Yes
Zhuhai Livzon	IgG + IgM	70.0 (70/100 TP) [60.0–78.8]	86.0 (86/100 TN) [77.6–92.1]	70.5 (43/61)	76.5 (26/34)	Yes
	IgG	52.0 (52/100 TP) [41.8–62.1]	100 (100/100 TN) [96.4–100]	52.5 (32/61)	64.7 (22/34)	Yes
	IgM	66.0 (66/100 TP) [55.9–75.2]	86.0 (86/100 TN) [77.6–92.1]	60.7 (37/61)	64.7 (23/34)	Yes

Specificity ranged from 74.3 to 99.1% for IgM/IgG, 82.9–100% for IgG only, and 75.2–98.0% for IgM only (Table 2, Fig. 1). For all tests, except for Beijing Wantai which is a combined IgM/IgG only, and Shenzhen Bioeasy where specificity for IgM/IgG and IgG were identical, IgG alone had a higher specificity than IgM/IgG (Table 2, Fig. 1). Four tests reported 100% specificity for IgG (Table 2). Nine LFAs had a higher IgG specificity than IgM (Table 2, Fig. 1).

## Diagnostic accuracy of LFAs against IgG ELISA

Of the 142 participants positive by RT-qPCR, 90 (63.4%) were positive by IgG ELISA. Of the 105 pre-pandemic samples, 85 were tested by IgG ELISA due to sample availability, of which 82 (96.5%) were negative by IgG ELISA. Compared to IgG ELISA, LFA sensitivity was found to range between 44.6% and 95.4% (Table 3). Shanghai Kehua had the highest sensitivity at 95.4% (Table 3). Compared to IgG ELISA, LFA specificity was found to range between 85.4% and 100% (Table 3). The highest agreement between IgG measured by LFA and IgG measured by ELISA was seen with Shanghai Kehua (Table 4).

**Table 3:** Sensitivity and specificity of LFAs against IgG ELISA.

Manufacturer	Sensitivity (%) [95% CI]	Specificity (%) [95% CI]
Beijing Wantai	92.3 (60/65 TP) [83.0–97.5]	98.8 (81/82 TN) [93.4–100]
Bionote	90.0 (54/60 TP) [79.5–96.2]	100 (78/78 TN) [95.4–100]
Core Technology	92.3 (60/65 TP) [83.0–97.5]	100 (82/82 TN) [95.6–100]
CTK Biotech	81.7 (49/60 TP) [69.6–90.5]	100 (76/76 TN) [95.3–100]
Edinburgh Genetics	80.0 (48/60 TP) [67.7–89.2]	100 (75/75 TN) [95.2–100]
GenBody	44.6 (29/65 TP) [32.3–57.5]	100 (76/76 TN) [95.6–100]
Jiangsu Bioperfectus	93.9 (61/65 TP) [85.0–98.3]	96.3 (79/82 TN) [89.7–99.2]
PRIME4DIA	86.7 (52/60 TP) [75.4–94.1]	100 (79/79 TN) [95.4–100]
Qingdao HIGHTOP	85.0 (51/60 TP) [73.4–92.9]	98.7 (78/79 TN) [93.2–100]
Shanghai Kehua	95.4 (62/65 TP) [87.1–99.0]	97.6 (80/82 TN) [91.5–99.7]
Shenzhen Bioeasy	83.1 (54/65 TP) [71.7–91.2]	85.4 (70/82 TN) [75.8–92.2]
Zhuhai Livzon	78.3 (47/60 TP) [65.8–87.9]	100 (78/78 TN) [95.4–100]

**Table 4:** Agreement and Cohen's Kappa between IgG measured by LFA and IgG ELISA.

	LFA	ELISA		Kappa [95% CI]
		Positive	Negative	
Beijing Wantai	Positive	60	1	0.9167 [0.8514–0.982]
	Negative	5	81	
Bionote	Positive	54	0	0.9105 [0.8405–0.9805]
	Negative	6	78	
Core Technology	Positive	60	0	0.9305 [0.8706–0.9904]
	Negative	5	82	
CTK Biotech	Positive	49	0	0.8327 [0.7379–0.9275]
	Negative	11	76	
Edinburgh Genetics	Positive	48	0	0.8163 [0.7171–0.9155]
	Negative	12	75	
GenBody	Positive	29	0	0.4733 [0.3238–0.6228]
	Negative	36	82	
Jiangsu Bioperfectus	Positive	61	3	0.9033 [0.8334–0.9732]
	Negative	4	79	
PRIME4DIA	Positive	52	0	0.8808 [0.8006–0.961]
	Negative	8	79	
Qingdao HIGHTOP	Positive	51	1	0.8510 [0.7620–0.9400]
	Negative	9	78	
Shanghai Kehua	Positive	62	2	0.9309 [0.8714–0.9904]
	Negative	3	80	
Shenzhen Bioeasy	Positive	54	12	0.6833 [0.5644–0.8022]
	Negative	11	70	
Zhuhai Livzon	Positive	47	0	0.8034 [0.7017–0.9051]
	Negative	13	78	

## Discussion

There is a plethora of LFAs available on the market today, all purporting to offer high sensitivity and specificity, but often without rigorous, manufacturer-independent evaluations. In this study, we evaluated 12 LFAs on serum samples collected from RT-qPCR -positive individuals and individuals with a wide range of diagnosed diseases pre-pandemic. We demonstrate the differences in sensitivity and specificity of the responses of combined IgM/IgG, IgG and IgM against a RT-qPCR and an IgG ELISA, in patients presenting with both acute and convalescent SARS-CoV-2 infections.

Bionote had the highest overall sensitivity (79.0% [95% CI: 69.7–86.5]), with a sensitivity of 88.2% at > 21 days post-symptom onset for an IgM/IgG response. Genbody Inc. had the lowest sensitivity with an overall sensitivity of 37.4% for an IgM/IgG response. Sensitivity of IgM/IgG and IgG improved for the majority of LFAs over 21 days post-symptom onset in agreement with other LFA evaluation studies.^9^ In this study, no test met the clinical sensitivity requirements of > 98% (95% CI: 96–100%) on samples collected ≥ 20 days post-symptom onset laid out in the target product profile (TPP) published by the UK government.^10^ However, our data are calculated on fewer than 200 confirmed positive cases as specified by the TPP.^10^ As expected with an earlier decline in IgM titres, fewer LFAs had improved sensitivity for IgM > 21 days post-symptom onset. It is important to note that the samples used in this study were collected before the roll out of any COVID-19 vaccine.

The large variation in performance in LFAs reported here is in accordance with other evaluations.^9^ The variations in diagnostic accuracy may in part be due to the antigen used to detect SARS-CoV-2 antibodies. The two main immunogenic antigens of SARS-CoV-2 are the nucleocapsid and the surface spike protein, split into domains S1 and S2, with the receptor-binding domain in S1. S1 is thought to be the most specific, with low-level cross-reactivity demonstrated for S2 and nucleocapsid.^11^ It is one or a combination of these antigens that are used for serological testing.

Not all manufacturers included in this evaluation disclose the antigen(s) used in their test. This information is key to testing during vaccine-rollout, with two vaccines, Pfizer-BioNTech and Moderna, containing mRNA encoding spike proteins to elicit an anti-spike immune response. LFAs that detect a response to spike antigens should prove useful for detecting both prior exposure to SARS-CoV-2, but also vaccinated individuals. Those that do not detect the relevant spike antigen may not prove as useful in detecting an immune response within vaccinated individuals but may have a role in identifying immune responses to breakout infections in vaccinated populations. Further studies should look at the use of LFAs in vaccinated individuals.

Our pre-pandemic negative panel consisted of serum from individuals diagnosed with a wide range of diseases, and in general, the false-positive results were found not to be linked to one disease. However, one sample from a returning traveler with malaria in 2005 gave a false positive result for 7 out of the 12 LFAs, which warrants further study. Of the 20 Influenza A samples, only 3 false positives were reported across all 12 LFAs. One sample with previous human coronavirus 229E exposure gave a false positive result for 4/12 LFAs as well as the EuroImmun IgG ELISA, and another gave a false positive result in 3/12 LFAs. Seven out of the twelve human coronavirus 229E and OC43 samples reported no false positives for any LFAs, whist 1 sample reported 2 false positives and the remaining 2 samples reported 1 false positive. This suggests cross-reactivity with immune responses to other human coronaviruses is possible but likely to be minimal, additionally pre-pandemic coronavirus patients often presented with other syndromes and coronavirus diagnosis has only been made due to multiplex molecular panels; this indicates samples used here may have unusual properties which may initiate cross-reaction as opposed to the coronaviruses present.

There is still no established ‘gold-standard’ serological test for SARS-CoV-2. The EuroImmun IgG ELISA was chosen at the time as it was one of the only CE-marked ELISA assays. We therefore use RT-qPCR as the main reference test in this study. Previous studies have reported false positives with the EuroImmun IgG ELISA, and we report here 3/105; one from a HIV patient in Nigeria in 2018, one from a patient with dengue virus in Brazil in 2015 and one with an individual with human coronavirus 229E in the UK in 2019. These were excluded from the ELISA/LFA specificity analysis. Cross-reactivity was seen in an evaluation of the EuroImmun Anti-SARS-CoV-2 NCP ELISA carried out in Nigeria in a negative control panel in which 50.2% of participants had the *P. falciparum* HRP2 antigen.^12^ The specificity of the EuroImmun ELISA in this study was 96.5%.

LFAs can be helpful in measuring exposure of a community to SARS-CoV-2, particularly in areas where testing of symptomatic individuals was not and is not readily available. Manufacturer-independent evaluations provide helpful data as to the accuracy of LFAs, as there is a large variation in the performance characteristics of these assays. Further evaluations are needed following the commencement of vaccination campaigns to evaluate the use of LFAs in vaccinated individuals.

## Declaration of Competing Interest

Emily R. Adams is Director of Epidemics and NTDs at Mologic.
